# Correction of the TNF-LT*α*-Deficient Phenotype by Bone Marrow Transplantation

**DOI:** 10.1155/1998/76963

**Published:** 1998

**Authors:** Bernhard Ryffel, Michel Le Hir, Mathias Müller, Hans-Pietro Eugster

**Affiliations:** 1 Institute of Pathology University of Basel Basel CH-4003 Switzerland; 2 Institute of Anatomy University of Zürich Zürich Switzerland; 3 Department of Immunology Medical School University of Cape Town Observatory 7925 South Africa

**Keywords:** TNF-LT*α* knockout, immunodeficiency, bone marrow transplantation, germinal centers, Ig class switch, alymphoplasia

## Abstract

Mice with inactivated TNF-LT*α* genes have profound abnormalities of the immune system with hypoimmunoglobulinaemia, lack of lymph nodes, undifferentiated spleen, and defective Ig class switch. Transplantation of bone marrow cells from wild-type mice restored the synthesis of TNF, corrected the splenic microarchitecture, repopulated the lamina propria with IgA-producing plasma cells, and normalized the serum immunoglobulin levels of TNF-LT*α* deficient mice. Furthermore, the formation of germinal centers in the spleen and the defective Ig class switch in response to a T-cell-dependent antigen is corrected. These data demonstrate that most TNFproducing cells are bone-marrow-derived, and that the immunodeficiency due to TNF-LT*α* deletion can be corrected to a large extent by normal bone marrow, cell transplantation.


*Developmental Immunology*, 1998, Vol. 6, pp. 253–261
Reprints available directly from the publisher
Photocopying permitted by license only

© 1998 OPA (Overseas Publishers Association)
N.V. Published by license under the
Harwood Academic Publishers imprint,
part of the Gordon and Breach Publishing Group
Printed in Malaysia

### Correction of the TNF-LT$\alpha$-Deficient Phenotype by Bone Marrow Transplantation

BERNHARD RYFFEL[a*], MICHEL LE HIR[b], MATHIAS MÜLLER[b] and HANS-PIETRO EUGSTER[b]

[a]*Institute of Pathology, University of Basel, CH-4003 Basel, Switzerland;* [b]*Institute of Anatomy, University of Zürich, Zürich, Switzlerland*

*(Received 5 August 1996; In final form 3 May 1997)*

Mice with inactivated TNF-LT$\alpha$ genes have profound abnormalities of the immune system with hypoimmunoglobulinaemia, lack of lymph nodes, undifferentiated spleen, and defective Ig class switch. Transplantation of bone marrow cells from wild-type mice restored the synthesis of TNF, corrected the splenic microarchitecture, repopulated the lamina propria with IgA-producing plasma cells, and normalized the serum immunoglobulin levels of TNF-LT$\alpha$ deficient mice. Furthermore, the formation of germinal centers in the spleen and the defective Ig class switch in response to a T-cell-dependent antigen is corrected. These data demonstrate that most TNF-producing cells are bone-marrow-derived, and that the immunodeficiency due to TNF-LT$\alpha$ deletion can be corrected to a large extent by normal bone marrow, cell transplantation.

*Keywords:* TNF-LT$\alpha$ knockout, immunodeficiency, bone marrow transplantation, germinal centers, Ig class switch, alymphoplasia

### INTRODUCTION

Tumor necrosis factor (TNF) and lymphotoxin $\alpha$ (LT$\alpha$) are mediators of inflammation and immune response (Aggarwal and Vilcek, 1992; Beutler, 1992). Both ligands form homotrimers and bind to two different receptors, tumor necrosis factor receptor 1 (TNFR-1), and TNFR-2 (Loetscher et al., 1990; Schall et al., 1990; Smith et al., 1993; Beutler and van Huffel, 1994). The genetic inactivation of the TNFR-1 (Pfeffer et al., 1993; Rothe et al., 1993) and TNFR-2 genes (Erickson et al., 1994) gave some indications on the *in vivo* effects of TNF and LT$\alpha$ mediated through these two receptor systems. Recently a third ligand, LT$\beta$, was identified that is expressed on the cell membrane as a LT$\alpha$/2LT$\beta$ heterotrimer and binds to the newly described LT$\beta$-specific receptor (LT$\beta$-R) (Androlewicz et al., 1992; Baens et al., 1993; Crowe et al., 1994; Force et al., 1995), suggesting a novel function for the LT$\alpha$/2LT$\beta$ heterotrimer. This pathway likely plays a role in the development of secondary lymphoid organs since LT$\alpha$-deficient mice

*Corresponding author. Present address: Department of Immunology, Medical School, University of Cape Town, Observatory 7925, South Africa.

lack lymph nodes (De Togni et al., 1994; Banks et al., 1995).

We have generated a TNF-LT$\alpha$-deficient mouse, which has a complete disruption of the TNF-LT$\alpha$ system, since it has neither of the two ligands signaling through the TNFR-1 and -2 nor through the LT$\beta$-R (Eugster et al., 1996). These TNF-LT$\alpha$ double-deficient mice develop normally with the exception of a profound immunodeficiency. The mutant mice have a sixfold increase of circulating B lymphocytes, no peripheral lymphoid organs, an undifferentiated spleen, a hypoimmunoglobulinemia, and very few IgA-producing plasma cells in the lamina propria of the small intestine.

Here, we asked to what extent the complex phenotype of TNF-LT$\alpha$ deficiency is due to incompetent lymphohemopoietic progenitors cells or rather due to a defective environment. For this, we attempted to restore the mutant phenotype by transplantation of wild-type bone marrow cells.

### RESULTS

### The Cells Producing TNF Systemically On Endotoxin Stimulation Are of Bone Marrow Origin

TNF-LT$\alpha$ double-deficient mice fail to produce TNF after endotoxin injection (Eugster et al., 1996). On transplantation with wild-type bone marrow cells, TNF-LT$\alpha$-deficient mice respond to endotoxin with TNF plasma levels at 1 h/z in the ng/ml range similar to wild-type mice (Fig. 1A). TNF-LT$\alpha$-deficient mice lethally irradiated and reconstituted with TNF-LT$\alpha$-deficient bone marrow cells served as controls and behaved essentially as nontransplanted TNF-LT$\alpha$-deficient mice. Conversely, lethally irradiated wild-type mice produced high levels of TNF when reconstituted with wild-type, but not with mutant bone marrow cells. These results demonstrate that the majority of cells producing TNF after endotoxin stimulation are radiosensitive and bone-marrow-

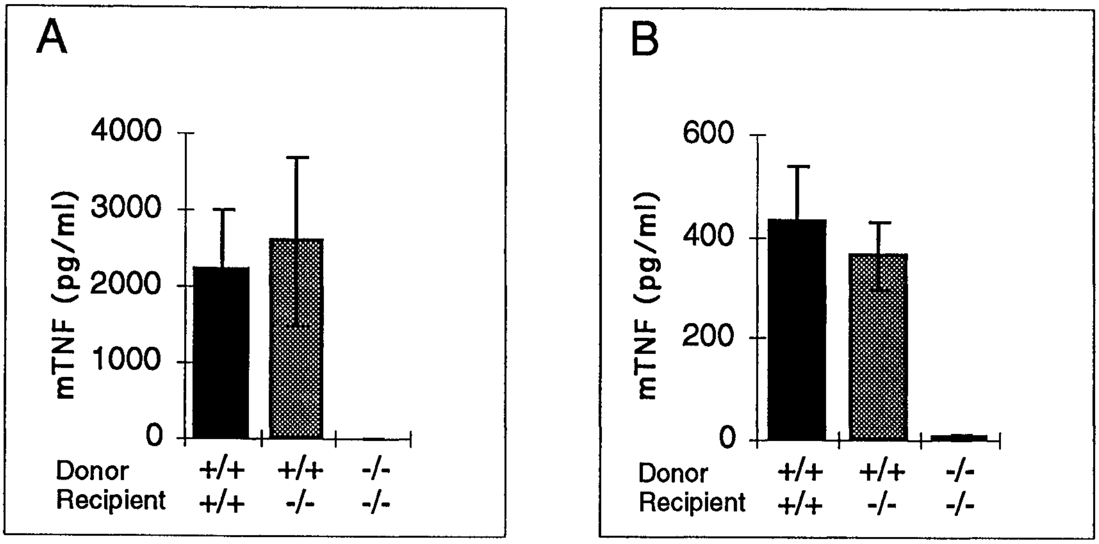

FIGURE 1 Correction of TNF production in TNF-LT$\alpha$ (−/−)-deficient mice on bone marrow transplantation from wild-type mice: (A) TNF plasma levels 1 hr after LPS injection. (B) *In vitro* TNF production by bone marrow-derived macrophages stimulated with LPS (3hr, 10 ng/ml) in the presence of IFN$\gamma$ (100 U). Bone-marrow cells (2 × $10^6$) from wild-type mice (+/+) or from TNF-LT$\alpha$ knockout (−/−) donor mice were transplanted into lethally irradiated wild-type (+/+) or TNF-LT$\alpha$ knockout recipient mice (−/−). Analysis of the *in vivo* TNF production in response to LPS (10 $\mu$g i.p.) was performed 14 weeks posttransplantation, at a time point where the hematological parameters were stabilized (A).

derived. *Ex vivo*, bone-marrow-derived macrophages from mutant mice reconstituted with wild-type donor cells produced similar amounts of TNF on LPS stimulation as those from wild-type controls (Figure 1B).

### Correction of Splenic Follicles, IgA-Producing Plasma Cell in the Lamina Propria and Serum Ig Levels of TNF-LT$\alpha$-Deficient Mice by Wild-Type Bone Marrow Transplantation

The spleen of TNF-LT$\alpha$-deficient mice are normal sized, but show no organization of the white pulp (Eugster et al., 1996). On transplantation of normal bone marrow cells, the architecture of the follicles of TNF-LT$\alpha$-deficient mice is corrected with a distinct separation of T- and B-cell areas (not shown) with appearance of MAdCAM-1 expression in the marginal sinus endothelium (not shown). As expected, mutant bone marrow was unable to correct the splenic structure of TNF-LT$\alpha$-deficient mice (data not shown). Whereas the correction of the spleen was successful, bone marrow transplantation did not reconstitute peripheral lymph nodes in TNF-LT$\alpha$-deficient mice, indicating a developmental defect in these mice.

TNF-LT$\alpha$-deficient mice have a profound reduction of serum immunoglobulin level, especially of IgA with an almost complete absence of IgA-producing plasma cells in the intestine. Upon wild-type marrow-cell transplantation, TNF-LT$\alpha$-deficient mice displayed a population of IgA-producing plasma cells in the lamina propria that was indistinguishable from that of wild-type controls (not shown). Since the splenic structure and the production of IgA in the lamina propria of the intestine were corrected by bone marrow transplantation, we asked whether the hypoimunoglobinaemia was also normalized. In agreement with previous studies, a profound reduction of Ig isotypes in TNF-LT$\alpha$-deficient mice was observed (Eugster et al., 1996). Figure 2A shows a marked reduction of IgG1 and almost complete absence of

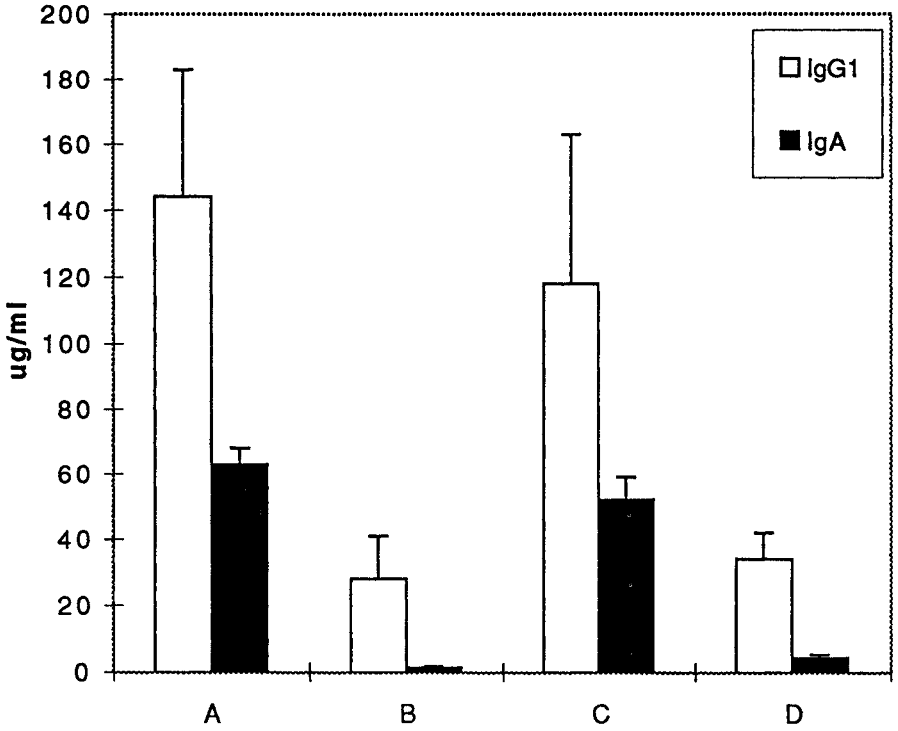

FIGURE 2 Serum IgG1 and IgA levels upon bone marrow reconstitution. (A) Wild-type control mice, (B) TNF-LT$\alpha$ knockout control mice; (C) transplantation of wild-type or (D) TNF-LT$\alpha$–/– bone marrow into TNF-LT$\alpha$ knockout mice ($n$ = 5 mice).

IgA in nontransplanted mutant mice. Upon normal bone marrow transplantation of TNF-LT$\alpha$-deficient mice, the IgG1 and IgA levels were increased reaching levels of wild-type mice (Figure 2C), where transplantation of mutant bone marrow did not correct the hypoimmunoglobulinemia (Figure 2D).

### Correction of Germinal Centers and Immunoglobulin Class Switch in TNF-LT$\alpha$-Deficient Mice by Wild-Type Bone Marrow Transplantation

Since the splenic follicles were reconstituted by bone marrow transplantation, we asked whether germinal centers are induced on immunization with the thymus-dependent antigen SRBC. Immunization induced the formation of germinal centers in bone-marrow-reconstituted TNF-LT$\alpha$-deficient mice (Figure 3). These secondary follicles displayed a distinct immunoreactivity with FDC-M1 antibody typical for follicular dendritic cells in germinal centers.

Furthermore, we tested whether the Ig class switch defect described previously in TNF-LT$\alpha$-deficient mice (Eugster et al., 1996) was restored after bone marrow transplantation. Upon transplantation of wild-type bone marrow, TNF-LT$\alpha$-deficient mice behaved like the wild-type controls, namely, they responded to SRBC by a transient synthesis of specific IgM peaking at day 6 and decreasing at day 15 (Figure 4A). At this time point, the IgG response started, as shown for IgG1, IgG2b, and IgG3 (Figs. 5B, 5C, and 5D). This was in contrast to the TNF-LT$\alpha$-deficient mice receiving mutant bone marrow, which showed no IgG responses on day 15, but persistent IgM titres on day 15 (Figs. 4A to 4D).

### DISCUSSION

The present experiments demonstrate that bone marrow cells are able to correct the TNF-LT$\alpha$ deficiency. Furthermore, the phenotype can be transferred into normal mice by bone transplantation from TNF-LT$\alpha$-deficient mice (Muller et al., 1996). Therefore, the genotype of the donor cells determines largely the phenotype of the irradiated host. Furthermore, the data suggest that the majority of cells producing TNF and LT$\alpha$ are radiosensitive. Several previous studies indicated that not only monocytes and T cells, but many other cell types, for example, Kupffer cells and hepatocytes, produce TNF and/or LT$\alpha$ (Aggarwal and Vilcek, 1992; Hunt et al., 1992; Vassalli, 1992).

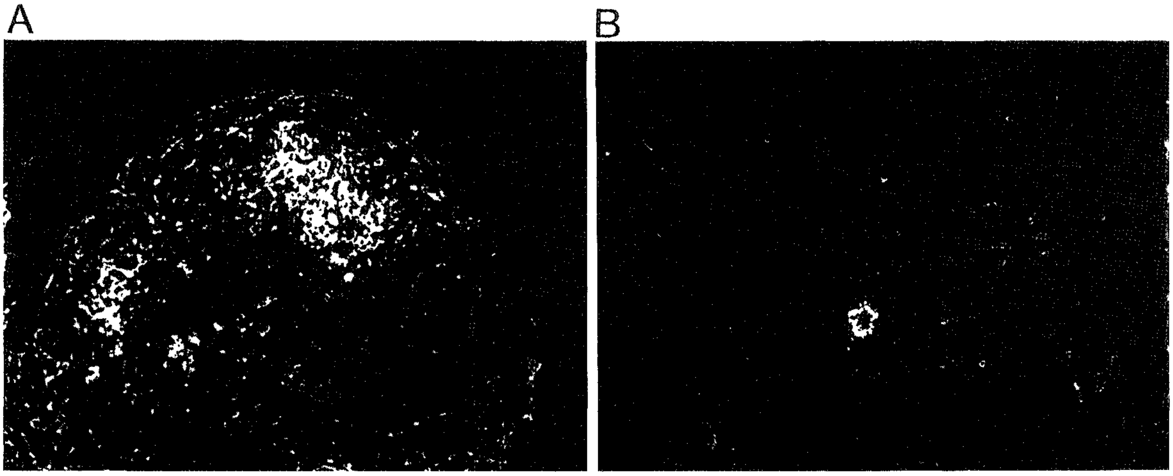

FIGURE 3 Induction of splenic germinal centers upon immunization in reconstitued TNF-LT$\alpha$ knockout mice by wild-type bone marrow transplantation. (A) FDC-M1 reactive follicular dendritic cells after transplantation of wild-type bone marrow cells ($2 \times 10^6$ cells) into irradiated TNF/LT$\alpha$ knockout recipient mice. (B) By contrast, wild-type mice transplanted with mutant bone marrow cells show no FDC-M1 reactivity typical of germinal centers

Kupffer cells are in part bone-marrow-derived, but are relatively radioresistant (Freudenberg et al., 1986). Our results suggest that the nonbone-marrow-derived cells apparently contribute little to the systemic release of TNF in response to endotoxin.

Together with the reconstitution of TNF and LT production, the splenic follicular structure was corrected, the lamina propria of the intestines repopulated by IgA-producing plasma cells, and the hypoimmunoglobulinemia restored.

Repeated immunization with antigen did not induce splenic germinal centers in TNF-LT$\alpha$-deficient mice. However, germinal centers were induced in TNF-LT$\alpha$-deficient mice reconstituted with normal bone marrow cells. A similar reconstitution of germinal centers by bone marrow cell transplantation was reported in LT$\alpha$-deficient mice (Matsumoto et al., 1996a). Therefore, TNF or LT$\alpha$ are necessary for the formation of germinal centers. Interestingly, TNF-R1-deficient mice lack also a germinal center response, suggesting that signaling through the type 1 TNF receptor is necessary for this effect (Le Hir et al., 1996; Matsumoto et al., 1996a). This finding is supported by our previous observation that follicular dendritic cells express high levels of TNF-R1 (Ryffel et al., 1991).

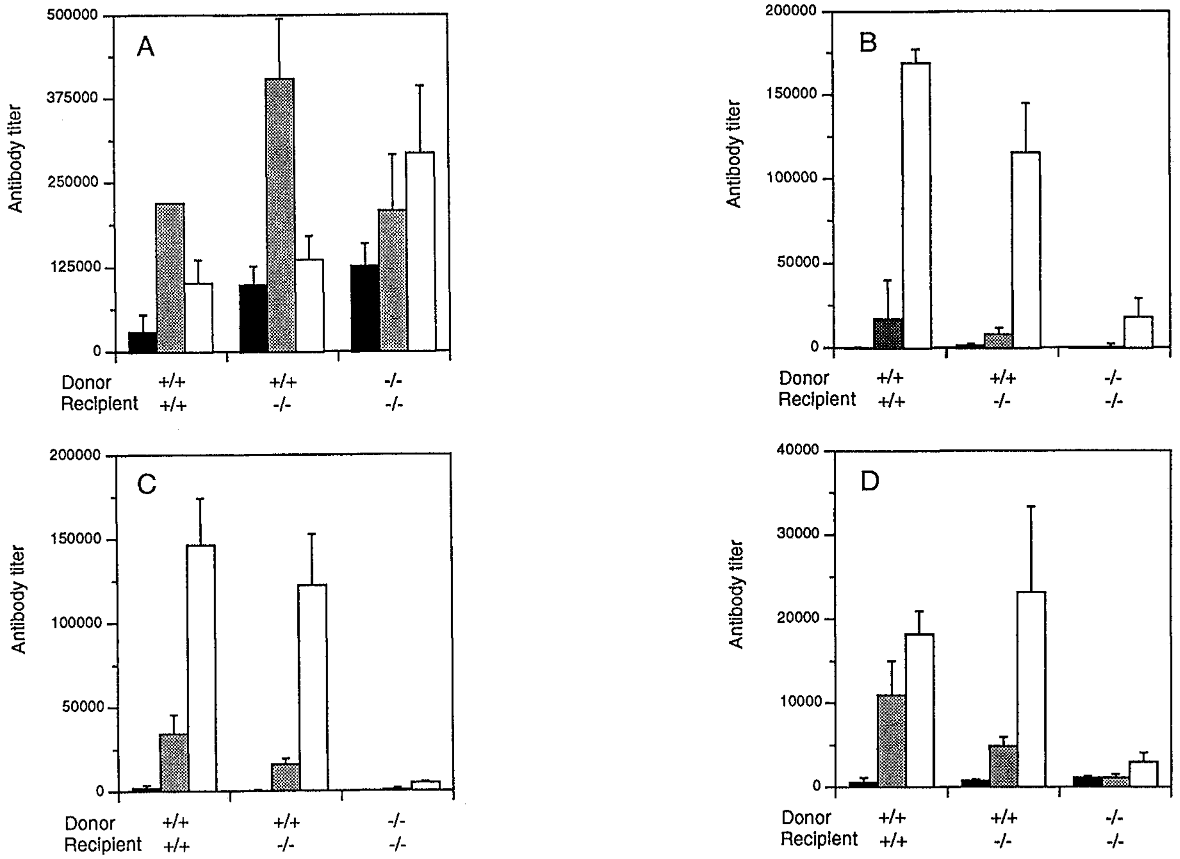

FIGURE 4 Restoration of antigen-induced immunoglobulin class switch upon bone marrow transplantation into TNF-LT$\alpha$-deficient mice. The reconstituted mice were immunized with SRBC ($2 \times 10^8$ i.p.) and the SRBC-specific (A) IgM, (B) IgG1, (C) IgG2b, and (D) IgG3 titers were determined by ELISA prior to (solid bar) and 6 days (shaded bar) and 15 days (white bar) after immunization. Results are pooled from (two) independent experiments and expressed as mean ± SEM ($n$ = 7 to 8 mice per group).

Although bone marrow transplantation from wild-type mice was able to restore the immunodeficiency of adult TNF-LT$\alpha$-deficient mice to a large extent, no lymph nodes were formed. Therefore, LT$\alpha$ is required during the embryonic development of lymph nodes. However, in the absence of TNF and LT$\alpha$, the spleen is formed although the microarchitecture is abnomal. These findings indicate that the spleen and lymph nodes have different requirements in embryonic development. Since LT$\alpha$-deficient mice (De Togni et al., 1994; Banks et al., 1995) also lack lymph nodes, but TNF-R-1 and -2 have normal lymph nodes (Pfeffer et al., 1993; Rothe et al., 1993; Erickson et al., 1994), the developmental pathways for lymph nodes require LT$\beta$-receptor signaling.

Several reports showed a possible involvement of soluble and membrane-bound TNF in immunoglobulin production. In particular, soluble TNF is able to enhance the immune response to SRBC (Ghiara et al., 1987). In addition it has been shown that TNF expressed on the membrane of $CD4^+$ T cells, together with the TNF-R1, participate in T-B cell interactions, resulting in enhanced immunoglobulin production (Aversa et al., 1993; Del-Prete et al., 1994).

Our data unequivocally demonstrate that TNF and/or LT$\alpha$ play a role in Ig class switching. Since no defect in the Ig class switch has been observed in TNF-R-1 and -2 deficient mice (Pfeffer et al., 1993; Rothe et al., 1993; Erickson et al., 1994), we propose that it is the signal from the LT$\alpha/\beta$ heteromer through the LT$\beta$-R that is necessary for isotype switching. Of note is also the finding that the defective Ig class switch was corrected by bone-marrow transplantation. Finally, germinal centers might not be necessary since a normal Ig class switch and affinity maturation was reported in LT$\alpha$-deficient mice on immunization at a high protein dose (Matsumoto et al., 1996a).

In conclusion, transplantation of normal bone marrow into TNF-LT$\alpha$ mutant mice allowed functional and to some extent morphological correction of the immune defect seen in the TNF-LT$\alpha$-deficient mice. Conversely, the mutant phenotype could be transferred by bone marrow cells transplanted into wild-type recipient mice.

### MATERIALS AND METHODS

### Mice

TNF-LT$\alpha$ knockout mice were generated as described on a mixed genetic 129/Ev/Sv $\times$ C57BL/6 background (Eugster et al., 1996). Seven- to ten-week-old wild-type and knockout mice bred in our animal facility and maintained under specific pathogen-free conditions were utilized.

### Reagents

Antibodies used in flow cytometry and FACS analysis were rat anti-mouse monoclonal antibodies (mAb) F4/80, B220, and CD3 (ATTC, Rockville, MD) and FDC-M1 (M. Kosco-Vilbois, Glaxo, Geneva). Anti-rat globulin goat IgG coupled to Cy-3 (Milan Analytica, LaRoche, Switzerland) or to phycoerythrin (Sigma, St. Louis) were used as a secondary antibody. Biotin-conjugated affinity-purified goat anti-mouse IgM, IgG1, IgG2a, IgG2b, IgG3, and IgA (Southern Biotechnology Associates, Birmingham, AL), and alkaline phophatase (AP)-coupled streptavidin (Jackson Immunoresearch Laboratories, West Grove, PA) were used in ELISA.

### Bone-Marrow Transplantation

Recipient mice received a lethal total-body irradiation (determined as 7.0 Gy in the knockout and wild-type mice), prior to the intravenous injection of $2 \times 10^6$ fresh, unseparated bone marrow cells.

### Injection of Endotoxin

Mice were injected intraperitoneally with 10 $\mu$g of LPS (*E. coli*, serotype O111:B4, Sigma, St. Louis) in saline solution (0.9%). Blood was taken at 1 h/z after LPS injection and plasma was analyzed for TNF levels. Blood samples were obtained from the tail vein, the retroorbital sinus, or cardiac puncture at euthanasia. Plasma concentrations of TNF were determined by a cytotoxicity assay using the WEHI-164 clone 13 cells (Espevik and Nissen-Meyer, 1986).

### Stimulation of Macrophages

Bone-marrow-derived macrophages were isolated from femurs and cultivated ($10^6$/ml) for 7 days in Dulbecco's minimal essential medium (DMEM) supplemented with 20% horse serum and 30% L929 cell-conditioned medium (as source of M-CSF). After 7 days culture, the cell preparation contains 99% macrophages (Kelso et al., 1982). The bone-marrow-derived macrophages were stimulated with LPS (10 ng/ml) for 3 h/z and the supernatant was assesssed for TNF activity as described.

### Anti-SRBC Serum Isotype and Ig Levels

Mice were immunized with $2 \times 10^8$ SRBC i.v. and serum was collected before and at 3, 6, 9, and 15 days postimmunization. Serum levels of anti-SRBC-specific Ig levels for IgM, IgG1, Ig2a, Ig2b, IgG3, and IgA were determined by a sandwich ELISA. Maxisorp microtiter plates were coated overnight with 50 $\mu$l of a solubilized extract (3 $\mu$g/ml) from SRBC prepared according to (Kelly et al. 1979). Thereafter, plates were blocked with 2% BSA containing PBS for 2 h/z at 37°C, and serial dilutions of the immune sera were incubated overnight at room temperature. Bound antibodies were detected with biotinylated goat anti-mouse Ig-isotype-specific antibodies (4 h/z at room temperature). Subsequently, streptavidin-conjugated to alkaline phosphatase and substrate were added for 45 min each, and the reaction was stopped with 1.5 M NaOH. Absorbance was read at 405 nm in a Titertek Multiskan spectrophotometer (Flow Laboratories). Serum titers are expressed as the reciprocal value of the dilution showing an optical density of 0.1 over background.

### Immunofluorescence Staining of Tissues

Tissues were snap frozen in supercooled (liquid nitrogen) isopentane and stored at −80°C, as previously described (Ryffel et al., 1991). Routinely prepared frozen sections were cut at 5 $\mu$m and fixed in acetone for 10 min, blocked in wash solution (50 mM Tris/HCl, 150 mM NaCl) for 30 min, and then incubated overnught at 4°C with rat anti-mouse mAb specific for CD3, B220, and FDC-M1. Controls, including incubation with secondary antibody alone, were uniformly negative. After two washes, sections were incubated with goat—anti-rat antibody labeled with Cy-3 at room temperature for 1 h/z. After a final wash, the slides were mounted in a Shandon Immunomount and coverslipped. Immunostained sections were examined with a Laser Scan Microscope 320 (Carl Zeiss, Zürich) in a confocal mode with a excitation wavelength of 543 Å.

### *References*

Aggarwal B.B. and Vilcek J. (1992). Tumor Necrosis Factor: Structure, Function, and Mechanism of Action, Vol. 56, Rose, N.R. ed. (New York: Marcel Dekker).

Androlewicz M.J., Browning J.L. and Ware C.F. (1992). Lymphotoxin is expressed as a heteromeric complex with a distinct 33-kDa glycoprotein on the surface of an activated human T cell hybridoma. J. Biol. Chem. **267**:2542-2547.

Aversa G., Punnonen J. and de-Vries J.E. (1993). The 26-kD transmembrane form of tumor necrosis factor alpha on activated CD4+ T cell clones provides a costimulatory signal for human B cell activation. J. Exp. Med. **177**:1575-1585.

Baens M., Chaffanet M., Cassiman J.J., van-den-Berghe H. and Marynen P. (1993). Construction and evaluation of a hncDNA library of human 12p transcribed sequences derived from a somatic cell hybrid. Genomics **16**:214-218.

Banks T.A., Rouse B.T., Kerley M.K., Blair P.J., Godfrey V.L., Kuklin N.K., Bouley D.M., Thomas J., Kanangat S. and Mucenski M.L. (1995). Lymphotoxin-a deficient mice. J. Immunol. **155**:1685-1693.

Beutler B. (1992). Tumour Necrosis Factor: The Molecules and their Emerging Role in Medicine (New York: Raven Press).

Beutler B. and van Huffel C. (1994). Unraveling function in the TNF ligand and receptor families. Science **264**:667-668.

Crowe P.D., VanArsdale T.L., Walter B.N., Ware C.F., Hession C., Ehrenfels B., Browning J.L., Din W.S., Goodwin R.G. and Smith C.A. (1994). A lymphotoxin-$\beta$-specific receptor. Science **264**:707-710.

De Togni P., Goellner J., Ruddle N.H., Streeter P.R., Fick A., Mariathasan S., Smith S.C., Carlson R., Shornick L.P., Strauss-Schoenberger J., Russel J.H., Karr R. and Chaplin D.D. (1994). Abnormal development of peripheral lymphoid organs in mice deficient in lymphotoxin. Science **264**:703-707.

Del-Prete G., De-Carli M., D'Elios M.M., Fleckenstein I.M., Fickenscher H., Fleckenstein B., Almerigogna F. and Romagnani S. (1994). Polyclonal B cell activation induced by herpesvirus saimiri-transformed human CD4+ T cell clones. Role for membrane TNF-alpha/TNF-alpha receptors and CD2/CD58 interactions. J. Immunol. **153**:4872-4879.

Erickson S.L., de Sauvage F.J., Kikly K., Pitts-Meek S., Gillett N., Sheehan K., Schreiber R.D., Goeddel D.V. and Moore M.W. (1994). Decreased sensitivity to tumour-necrosis factor but normal T-cell development in TNF receptor-2-deficient mice. Nature **372**:560-563.

Espevik T. and Nissen-Meyer J. (1986). A highly sensitive cell line, WEHI 164 clone 13, for measuring cytotoxic factor/tumor necrosis factor from human monocytes. J. Immunol. Meth. **95**:99-105.

Eugster H.P., Muller M., Karrer U., Car B.D., Schnyder B., Eng V.M., Woerly G.M.L.H., Di Padova F., Aguet M., Zinkernagel R., Bluethman H. and Ryffel B. (1996). Multiple immune abnormalities in tumor necrosis factor and lymphotoxina double deficient mice. Int. Immunol. **8**:23-36.

Force W.R., Walter B.N., Hession C., Tizard R., Kozak C.A., Browning J.L. and Ware C.F. (1995). Mouse lymphotoxin-beta receptor. Molecular genetics, ligand binding, and expression. J. Immunol. **155**:5280-5288.

Freudenberg N., Freudenberg M.A., Hoess C.D., Schrecker H. and Galanos C. (1986). Investigation into the origin of mouse liver sinusoidal cells. Virchows Arch. A Pathol. Anat. Histopathol. **410**:1-7.

Ghiara P., Boraschi D., Nencioni L., Ghezzi P. and Tagliabue A. (1987). Enhancement of in vivo immune response by tumor necrosis factor. J. Immunol. **139**:3676-3679.

Hunt J.S., Chen H.L., Hu X.L., Chen T.L. and Morrison D.C. (1992). Tumor necrosis factor-$\alpha$ gene expression in the tissues of normal mice. Cytokine **4**:340-346.

Kelly B.S., Levy J.G. and Sikora L. (1979). The use of the enzyme-linked immunosorbent assay (ELISA) for the detection and quantification of specific antibody from cell cultures. Immunology **37**:45-52.

Kelso A., Glasebrook A.L., Kanagawa O. and Brunner K.T. (1982). Production of macrophage-activating factor by T lymphocyte clones and correlation with other lymphokine activities. J. Immunol. **129**:550-561.

Le Hir M., Bluethmann H., Kosco-Vilbois M.H., Müller M., di Padova F., Ryffel B. and Eugster H.P. (1996). Differentiation of follicular dendritic cells and full antibody response requires tumor necrosis factor receptor-1 signaling. J. Exp. Med. **183**:2367-2372.

Loetscher H., Pan Y.C., Lahm H.W., Gentz R., Brockhaus M., Tabuchi H. and Lesslauer W. (1990). Molecular cloning and expression of the human 55 kd tumor necrosis factor receptor. Cell **61**:351-359.

Matsumoto M., Lo S.F., Carruthers C.J., Min J., Marithasan S., Huang G., Plas D.R., Martin S.M., Geha R.S., Nahm M.H. and Chaplin D.D. (1996a). Affinity maturation without germinal centres in lymphotoxin-a-deficient mice. Nature **382**:462-466.

Matsumoto M., Nariathasan S., Hahm M.H.F.N., Peschon J.J. and Chaplin D.D. (1996b). Role of lymphotoxin and type 1 TNF receptor in the formation of germinal centers. Science **271**:1289-1291.

Muller M., Eugster H.P., Le Hir M., Shakhov A., Di Padova F., Maurer C., Quesniaux V.F.J. and Ryffel B. (1996). Correction and transfer of immunodeficiency due to TNF-LTa deletion by bone marrow transplantation. Mol. Med.

Pfeffer K., Matsuyama T., Kundig T.M., Wakeham A., Kishihara K., Shahinian A., Wiegmann K., Ohashi P.S., Kronke M. and Mak T.W. (1993). Mice deficient for the 55 kd tumor necrosis factor receptor are resistant to endotoxic shock, yet succumb to L. monocytogenes infection. Cell **73**:457-467.

Rothe J., Lesslauer W., Lotscher H., Lang Y., Koebel P., Kontgen F., Althage A., Zinkernagel R., Steinmetz M. and Bluethmann H. (1993). Mice lacking the tumour necrosis factor receptor 1 are resistant to TNF-mediated toxicity but highly susceptible to infection by Listeria monocytogenes. Nature **364**:798-802.

Ryffel B., Brockhaus M., Durmuller U. and Gudat F. (1991). Tumor necrosis factor receptors in lymphoid tissues and lymphomas. Source and site of action of tumor necrosis factor alpha. Amer. J. Pathol. **139**:7-15.

Schall T.J., Lewis M., Koller K.J., Lee A., Rice G.C., Wong G.H., Gatanaga T., Granger G.A., Lentz R., Raab H., et al. (1990). Molecular cloning and expression of a receptor for human tumor necrosis factor. Cell **61**:361-370.

Smith C.A., Gruss H.J., Davis T., Anderson D., Farrah T., Baker E., Sutherland G.R., Brannan C.I., Copeland N.G., Jenkins N.A., et al. (1993). CD30 antigen, a marker for Hodgkin's lymphoma, is a receptor whose ligand defines an emerging family of cytokines with homology to TNF. Cell **73**:1349-1360.

Vassalli P. (1992). The pathophysiology of tumor necrosis factor. Annu. Rev. Immunol. **10**:411-452.

